# Identification of a prognostic cuproptosis-related signature in hepatocellular carcinoma

**DOI:** 10.1186/s13062-023-00358-w

**Published:** 2023-02-07

**Authors:** Yuqiao Chen, Lu Tang, Wentao Huang, Fakolade Hannah Abisola, Youyu Zhang, Gewen Zhang, Lei Yao

**Affiliations:** 1 Department of General Surgery, Xiangya Hospital, Central South University, Changsha, Hunan China; 2 Department of Anesthesiology, Xiangya Hospital, Central South University, Changsha, Hunan China; 3 Department of Thoracic Surgery, Xiangya Hospital, Central South University, Changsha, Hunan China; 4 National Clinical Research Center for Geriatric Disorders, Xiangya Hospital, Central South University, Changsha, China

**Keywords:** Hepatocellular carcinoma, Cuproptosis, Prognostic signature, Bioinformatic analysis, PDXK, Metastasis

## Abstract

**Background:**

Cuproptosis is a new type of copper-induced cell death that is characterized by the aggregation of lipoylated tricarboxylic acid (TCA) cycle proteins. However, its role in hepatocellular carcinoma (HCC) remains unclear. The goal of this research is to develop a cuproptosis-related signature predicting the prognosis of HCC.

**Methods:**

The cuproptosis-related genes were defined using Pearson correlation coefficients. LASSO-Cox regression analysis was used to evaluate the prognostic values of cuproptosis-related genes to construct a cuproptosis-related prognostic model. The immune microenvironment analysis was performed by “ssGSEA” to reveal the associated immune cell infiltration patterns with the cuproptosis-related genes signature. The expression levels of one of the prognostic genes PDXK were then verified in HCC samples by Western Blot and immunohistochemistry. The potential roles of target genes in cuproptosis were further explored during in-vitro experiments.

**Results:**

A total of 136 cuproptosis-related genes were discovered using Pearson correlation analysis in HCC. A cuproptosis-related signature that included 5 cuproptosis-related genes (PDXK, HPN, SLC25A28, RNFT1, CLEC3B) was established in the TCGA-LIHC training cohort. TCGA validation cohort and another two external validation cohorts confirmed the robustness of the signature’s predictive value. Moreover, a nomogram using the risk score was created to best predict the survival of HCC patients. The immune microenvironment analysis revealed distinct immune infiltrations patterns between different risk groups based on the signature model. Furthermore, the upregulation of PDXK was confirmed in HCC tumor tissues in 30 clinical HCC specimens. The knockdown of PDXK reduced the proliferation, migration and invasion of HCC cells. Besides, the expression of PDXK was upregulated after the induction of cuproptosis by elesclomol–CuCL_2_, which could be suppressed when pretreated with a copper ion chelator. And PDXK deficiency increased the sensitivity of HCC cells to cuproptosis inducer.

**Conclusion:**

Our study identified a new cuproptosis-related gene signature that could predict the prognosis of HCC patient. Besides, the upregulated PDXK could promote the proliferation and metastasis of HCC. And PDXK deficiency facilities cuproptosis in HCC. Therefore, these fundings highlighted that PDXK might serve as a potential diagnostic and therapeutic target for HCC.

**Supplementary Information:**

The online version contains supplementary material available at 10.1186/s13062-023-00358-w.

## Introduction

Liver cancer is one of the most lethal cancers with cancer-related death accounting for nearly 30,000 deaths per year [1]. Hepatocellular carcinoma (HCC) is the most common type of liver cancer, accounting for 70–85% of all cases. It usually develops in the context of advanced chronic liver disease, which is caused by the alcohol abuse and the affection of hepatitis B virus (HBV) or hepatitis C virus (HCV) [2]. Despite the rising occurrence of liver cancer, there remains a startling paucity of therapy options and the patient’s chances of survival are dismally poor, with a 5-year survival, being merely 18% [3]. Thus, research into reliable and promising prognostic biomarkers for HCC patients is crucial.

Recently, a group-breaking study by Tsvetkov et al*.* demonstrated a novel type of cell death, copper-dependent cell death termed cuproptosis [4]. In contrast to all other known cell death mechanisms, such as apoptosis [5], necroptosis [6], autophagy [6], pyroptosis [7], and ferroptosis [8], this kind of copper toxicity refers to a previously unidentified cell death mechanism. Cuproptosis is characterized by aggregation of lipoylated tricarboxylic acid (TCA) cycle proteins, which leads to destabilization of Fe–S cluster proteins, and increased proteotoxic stress [4]. More specifically, cuproptosis occurs when Cu levels rise and is initiated by FDX1. FDX1 encodes a Cu2 + reductase, which induces mitochondrial protein lipoylation including dihydrolipoamide S-succinyltransferase (DLST), dihydrolipoamide S-acetyltransferase (DLAT), dihydrolipoamide branched chain transacylase E2 (DBT), and glycine cleavage system protein H (GCSH). These lipoylated protein aggregation and Fe–S cluster protein destabilization result in proteotoxic stress and cell death. FDX1 is a direct target of elesclomol (ES), which is a copper ionophore that transports copper into cells. Besides, Buthionine sulfoximine (BSO) could promote cuproptosis by depleting GSH which inhibits cuproptosis by acting as a thiol-containing copper chelator. It is anticipated that cuproptosis will become a crucial target for the treatment of cancer.

The tumor immune microenvironment (TME) of HCC is formed by a complex cellular composition in which different types or subtypes of myeloid cells and lymphocytes play important roles in inflammation, tumor immune evasion, and immunotherapeutic response [9]. Although the immune microenvironment of HCC is poorly characterized [10, 11]. The disorder and imbalance of TME in HCC caused by various factors may be one of the most critical mechanisms for its development and progression [12, 13]. But, whether the cuproptosis is involved in the remodeling of TME and has some impact on tumor progression and immunotherapy efficacy in HCC is still obscure.

The purpose of this study was to find predictive cuproptosis-related genes in HCC that can not only provide valuable insight into the molecular networks, the signaling pathway and the tumor immune infiltration that is related to cuproptosis in HCC, but can also be used to identify HCC patients at high risk of poor survival.

## Methods

### Data retrieval and acquisition of cuproptosis-related genes

The transcriptome data and clinical information of TCGA-LIHC and ICGC-LIHC were downloaded from UCSC XENA (https://xenabrowser.net/) and the Hepatocellular carcinoma database (HCCDB, http://lifeome.net/database/hccdb/) [14], respectively. GSE54236, GSE10143, GSE144269, and GSE76427 were derived from the Gene Expression Omnibus (GEO) database (https://www.ncbi.nlm.nih.gov/geo/).

The 36 cuproptosis genes were extracted from a previous study [4]. The cuproptosis-related genes were then defined using Pearson correlation coefficients. The cuproptosis-related genes were determined using a p-value of less than 0.001 and a Pearson correlation coefficient absolute value of larger than 0.3 (|R|> 0.3).

### The identification and validation of a prognostic cuproptosis-related signature

The cuproptosis-related genes having prognostic values in the training group were first discovered using univariate cox regression. Secondly, using the R package “glmnet” and the least absolute shrinkage and selection operator (LASSO) cox regression analysis, a predictive gene signature was created. This signature’s risk score was calculated as follows:

$$ \begin{aligned} r{\text{isk}}\,{\text{score}} & = ({\text{expr}}_{gene1} \, \times \,{\text{coefficient}}_{gene1} ) \\ & \quad + ({\text{expr}}_{gene2} \, \times \,{\text{coefficient}}_{gene2} ) \\ & \quad + \cdots + \,({\text{expr}}_{geneX} \, \times \,{\text{coefficient}}_{geneX} ). \\ \end{aligned} $$

TCGA validation cohort and two external cohorts (ICGC and GSE54236) were further used to validate the prognostic efficacy of the cuproptosis-related genes signature. All patients were separated into two groups based on the median of risk score: high-risk and low-risk. The R programs “survival” and “survminer” were used to examine the Kaplan–Meier curves of the two groups of patients. A time-dependent ROC curve analysis was then carried out, using the “survivalROC” R program to test the predictive accuracy of the cuproptosis-related genes signature. The prediction ability was assessed using the area under the curve (AUC).

### The construction and validation of the nomogram

Univariate cox regression analysis was used to assess the relationship between risk factors (based on risk score, age, gender, T-stage, N-stage, M-stage, and NCCN stage) and prognosis. Followed by the multivariate cox regression analysis to examine if the risk score of the signature and clinical characteristics were independent predictors of overall survival (OS). A nomogram was created using the risk score and other clinical indicators to predict the 1-, 3-, and 5-year OS of HCC patients. We used data calibration curves to determine the nomogram’s predictive accuracy. These curves were built to see if the predicted and observed OS probabilities were in accord. To measure the nomogram’s capacity to differentiate and predict, the concordance index (C-index) was also determined. The C-index ranged from 0.5 to 1.0, with a higher C-index indicating stronger differentiating ability of the predictive model.

### Functional enrichment analysis and immune cell infiltration analysis

The biological processes (BP), molecular functions (MF), and cellular components (CC) of differentially expressed genes between high-risk and low-risk groups based on the cuproptosis-related signature were determined using Gene Ontology (GO) analysis. Additionally, the Kyoto Encyclopedia of Genes and Genomes (KEGG) pathway analysis and Gene set variation analysis (GSVA) was used to investigate the pathways involved in differentially expressed genes in various risk groups [15]. The gene sets of hallmarkers were obtained from the Molecular Signatures Database (MSigDB, http://software.broadinstitute.org/gsea/msigdb/index.jsp) for GSVA analysis. The ssGSEA algorithm was used to evaluate the patterns of the tumor immune infiltrations [16].

### Cell lines, RNA interfering and western bolt

Cells were cultured in DMEM culture medium, supplemented with 10% FBS in a standard humidified incubator with 5% CO2 at 37 °C. The expression of PDXK in different HCC cell lines were tested by Western Blot. Two cell lines with the highest expression of PDXK were chosen for further experiment. The knockdown of PDXK in HCC cells was achieved via the transfection of the PDXK specific small Interfering RNA (siRNA) using Lipofectamine 3000 reagent (Invitrogen, Massachusetts, USA) according to the manufacturer’s protocol. The PDXK specific Small Interfering RNA were synthesized from GenePharma (Shanghai, China) and the sequences of siRNAs are as the following: si-PDXK-1: 5ʹ-GGUGCCGCUUGCAGACAUUTT AAUGUCUGCAAGCGGCACCTT-3ʹ; si-PDXK-2: 5ʹ-GGGCAGCAACUACCUGAUUTT AAUCAGGUAGUUGCUGCCCTT-3ʹ. The knockdown efficiency was evaluated by Western blotting and Real Time quantitative PCR (RT-qPCR) after 48 h transfection. For the western blotting, RIPA buffer was used to prepare whole cell lysates, and proteins were separated with SDS/PAGE gel before being transferred to PVDF membranes and incubated overnight with corresponding primary antibodies. Following that, HRP-conjugated secondary antibodies were incubated, and ECL was used to detect chemiluminescent signals. Antibodies and primers and were listed in Additional file 1: Table S2 and Additional file 2: Table S3.

### Migration, invasion, and proliferation assay

For in vitro migration and invasion assay, the 24-well transwell chambers (Transwell, Corning Costar) were employed. For in vitro migration assays, a total of 4 × 10^4^ cells were added to the upper chamber (Transwell, Corning Costar) after being suspended in serum-free medium. The lower chamber was filled with the medium of 20% FBS. For in vitro invasion assays, the upper membranes were coated with 40 μL matrigel (Matrigel™ GFR Membrane Matrix, #356231, Corning, USA) in advance and a total of 8 × 10^4^ cells were seeded to the upper chamber. The cells in the upper chamber were carefully cleaned with a cotton swab after 24 h culture. The cells attached to the filter’s lower surface were fixed with 4% paraformaldehyde and stained with crystal violet. The cells on the lower surface of the membrane filter were captured on camera under a microscope.

For wound healing assay, cells (1 × 10^5^ cells/well) were seeded into 6-well plates. After the formation of adherent confluent cell monolayer, the cancer cells were starved for 8 h. Wounds were created by a 10 μL pipette. The wound was inflicted at 0 h, and wound was photographed after 24 h. ImageJ was used to calculate the percentage of areas covered by migrated cells.

Colony formation assays were used to determine cell viability. Cells were seeded at a density of 6 × 10^2^ cells/plate in 6-well culture plates. The cells were cultured in normal medium for 10 days after transduction of PDXK specific siRNA or random control. Surviving tumor cells were fixed with 4% paraformaldehyde and stained with crystal violet before counting the colonies. MTT (Sigma) assays were performed as described previously [17].

### Clinical specimens and immunohistochemistry (IHC)

A total of 30 HCC tumor tissue with their adjacent non-cancerous lung tissues were obtained from Xiangya Hospital, Central South University. The ethics committee of the Xiangya Hospital of Central South University gave its approval to this study. Written informed consent was obtained from each patient to use his/her material.

Slices were dewaxed and antigen retrieved. Following the blocking step with bovine serum albumin, the slices were incubated overnight at 4 °C with the primary antibodies against PDXK (1:200) and were washed thrice with PBS. After 30 min of incubation with the secondary antibody at 37 °C, the slices were treated with DAB color rendering for 5–10 min and hematoxylin redye for 10 s. Finally, the slices were examined under a microscope. Immunohistochemical staining was calculated based on staining intensity and quantity. The staining intensity was ranked as negative, weak, moderate, and strong (0, 1, 2, and 3). The staining quantity was calculated based on the percentage of stained cells, which was ranked by < 10%, 10–25%, 25–50%, 50–75%, and > 75% (0, 1, 2, 3, and 4). The IHC score was calculated by staining intensity × quantity, IHC score ≤ 6.0 was defined as low expression and > 6.0 was defined as high expression [18].

### Statistical analysis

All statistical analyses were performed using Prism (Version 9.0) and R (Version 4.0.3) software. The statistical significance was determined using the 0.05 *p*-value, and all *p*-values were two-tailed.

## Results

### Identification of the cuproptosis related genes in HCC

**Fig. 1:**
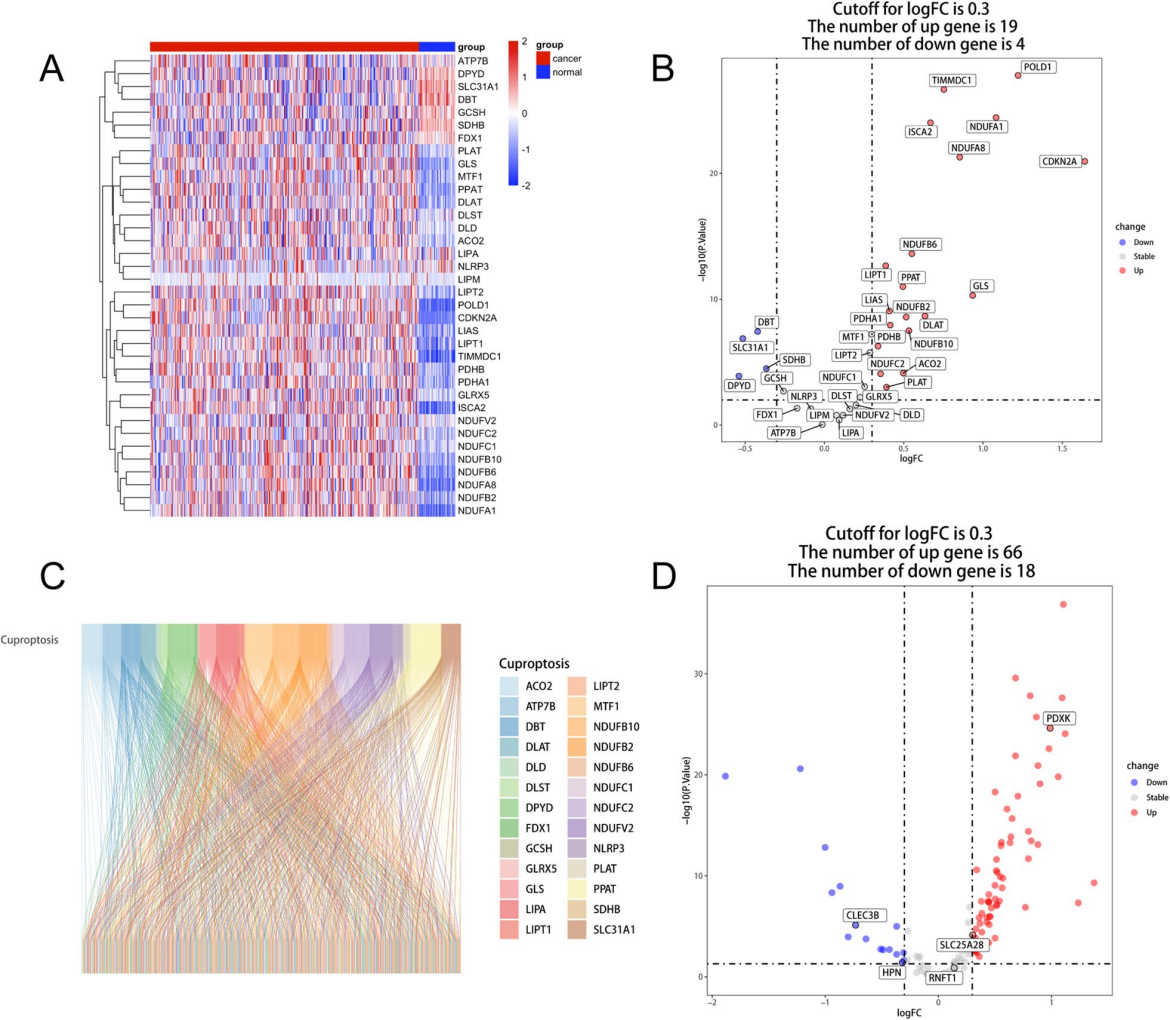
The determination of cuproptosis-related genes in HCC. **A** The heatmap representing the previous reported 36 cuproptosis genes in HCC normal and tumor tissue. **B** The volcano map depicting the differential expressed patterns of cuproptosis genes in HCC normal and tumor tissue. **C** The cuproptosis-related genes in the HCC were visualized using a Sankey diagram. **D** the volcano plot depicted the expression patterns of cuproptosis-related genes in TCGA-LIHC

In HCC samples, 19 Cuproptosis genes were upregulated and 4 genes were downregulated, indicating that cuproptosis in HCC was dysregulated (Fig. 1A, B). By the Pearson relation analysis, 136 cuproptosis-related genes were linked to the cuproptosis genes (Additional file 3: Table S1), which were depicted using a Sankey diagram (Fig. 1C). The following differential analysis revealed that 66 cuproptosis-related genes were upregulated in tumor tissue, whereas 18 were downregulated (Fig. 1D).

### Construction and validation of cuproptosis-related signature

**Fig. 2:**
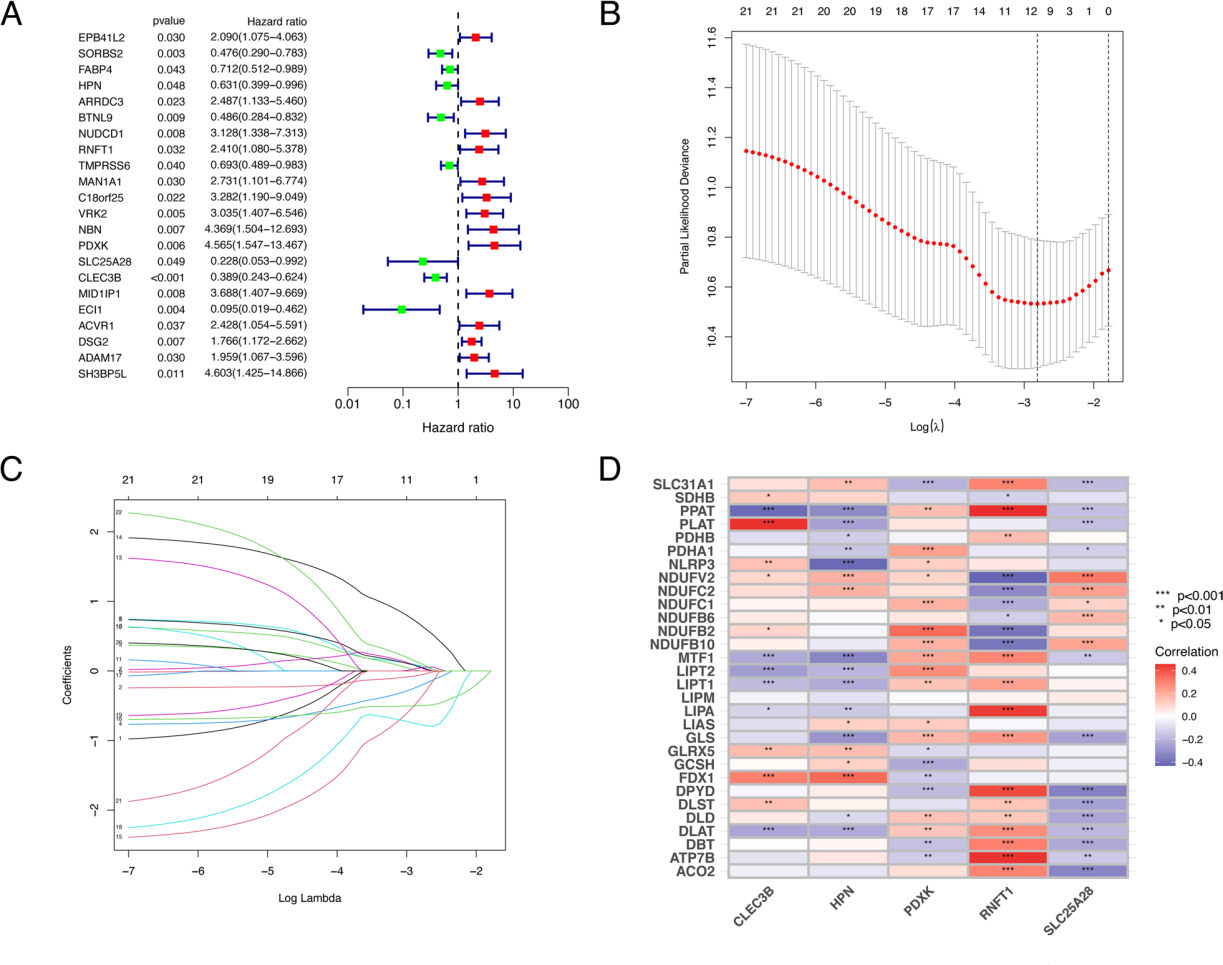
Construction of a cuproptosis-related predictive model. **A** The forest plot demonstrated the hazard ratio of 22 cuproptosis-related genes with prognostic values filtered by the univariate cox regression analysis. **B**–**C** The cuproptosis-related genes with prognostic values were subjected to LASSO Cox regression analysis. Five cuproptosis-related genes were screened to build the predictive model for survival, including PDXK, HPN, SLC25A28, CLEC3B, and RNFT1. **D** This heatmap depicted relationship between the five Cuproptosis-related prognostic genes with the previous reported cuproptosis genes

Using a univariate cox regression analysis, 22 prognostic cuproptosis-related genes were identified among the 84 differential expressed cuproptosis-related genes (Fig. 2A). The cuproptosis-related signature was explored using the LASSO cox regression analysis (Fig. 2B, C). The heatmap demonstrated the relationship between the cuproptosis gene and five prognostic cuproptosis-related genes, including PDXK, HPN, SLC25A28, RNFT1, and CLEC3B (Fig. 2D). This signature’s risk score was calculated as follows: Risk score: (− 0.5910043 × HPN) + (0.5342352 × RNFT1) + (0.7706872 × PDXK) + (− 1.0743333 × SLC25A28) + (− 0.6488743 × CLEC3B).

**Fig. 3:**
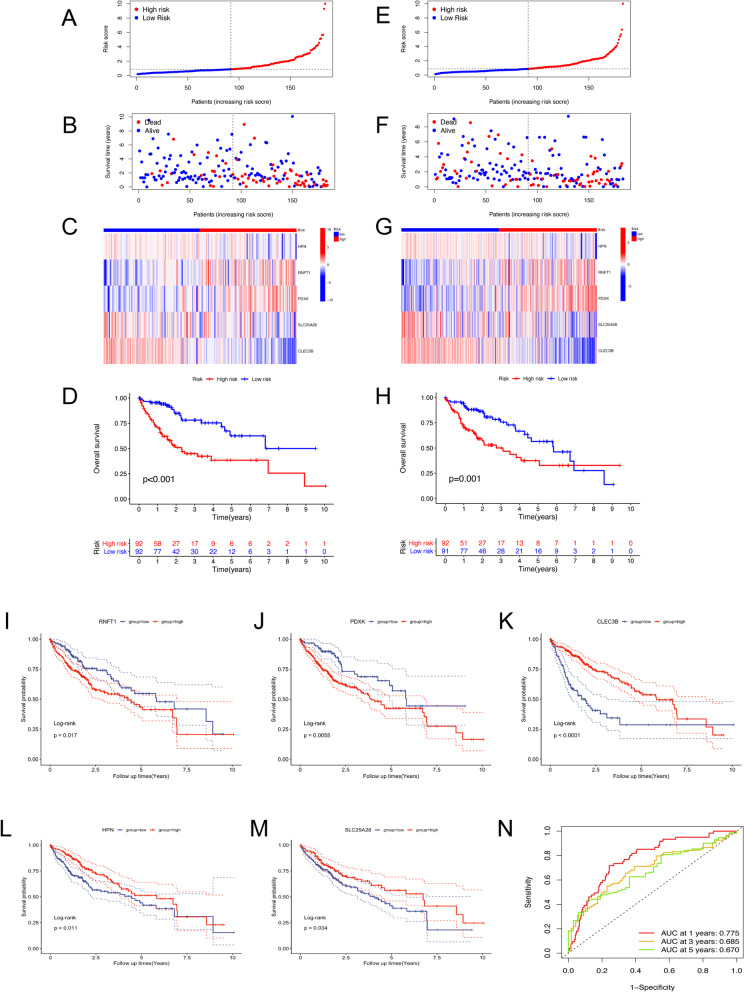
The risk model of five cuproptosis-related gene in TCGA training and validation cohorts. **A**, **E** In the TCGA training and testing cohort, the risk score distribution and median value are shown. **B**, **F** The dot plot depicted the TCGA training and testing cohort’s survival status, survival time, and risk score distributions. **C**, **G** In the TCGA training and testing cohort, a heatmap shows the patients were divided into high- and low-risk groups according to the risk factor of five cuproptosis-related gene expression. **D**, **H** In the TCGA training and testing cohort, Kaplan–Meier showed the survival of patients in high- and low-risk groups. **I**–**M** The results of survival analysis in TCGA-LIHC revealed that the high expression of CLEC3B, HPN, and SLC25A28 suggested longer survival periods. Higher levels of RNFT1 and PDXK expression, on the other hand, suggested a shorter survival time. **N** For 1-, 3-, and 5-year overall survival, the AUC of the survival ROC curves was utilized to assess the predictive efficacy of the developed risk signature

Based on the median risk score, the patients in TCGA training cohort were further stratified as high-risk (N = 92) or low-risk (N = 92) (Fig. 3A–C). Patients in the high-risk group had a significantly shorter overall survival than those in the low-risk group (Fig. 3D, *p* < 0.001). To further validate the robustness of prognostic value of this signature, the patients in the TCGA validation cohort (N = 183) were also separated into high-risk (N = 92) and low-risk (N = 91) groups (Fig. 3E–G). Consistent with the results of training cohort, high-risk patients had a shorter survival time than low-risk patients in validation cohort (Fig. 3H, *p* = 0.001).

Separately, the survival curve with the log-rank test revealed that high RNFT1 and PDXK expression indicated a worse clinical outcome (Fig. 3I,J), whereas higher levels of CLEC3B, HPN, and SLC25A28 expression were associated with a longer overall survival in TCGA-LIHC dataset (Fig. 3K–M). At 1-year, at 2-years, and at 5-years, the AUC of the ROC curve for risk variables was 0.775, 0.685, and 0.670, respectively (Fig. 3N) in TCGA-LIHC cohort, which indicated a relatively high predictive accuracy for HCC patients.

**Fig. 4:**
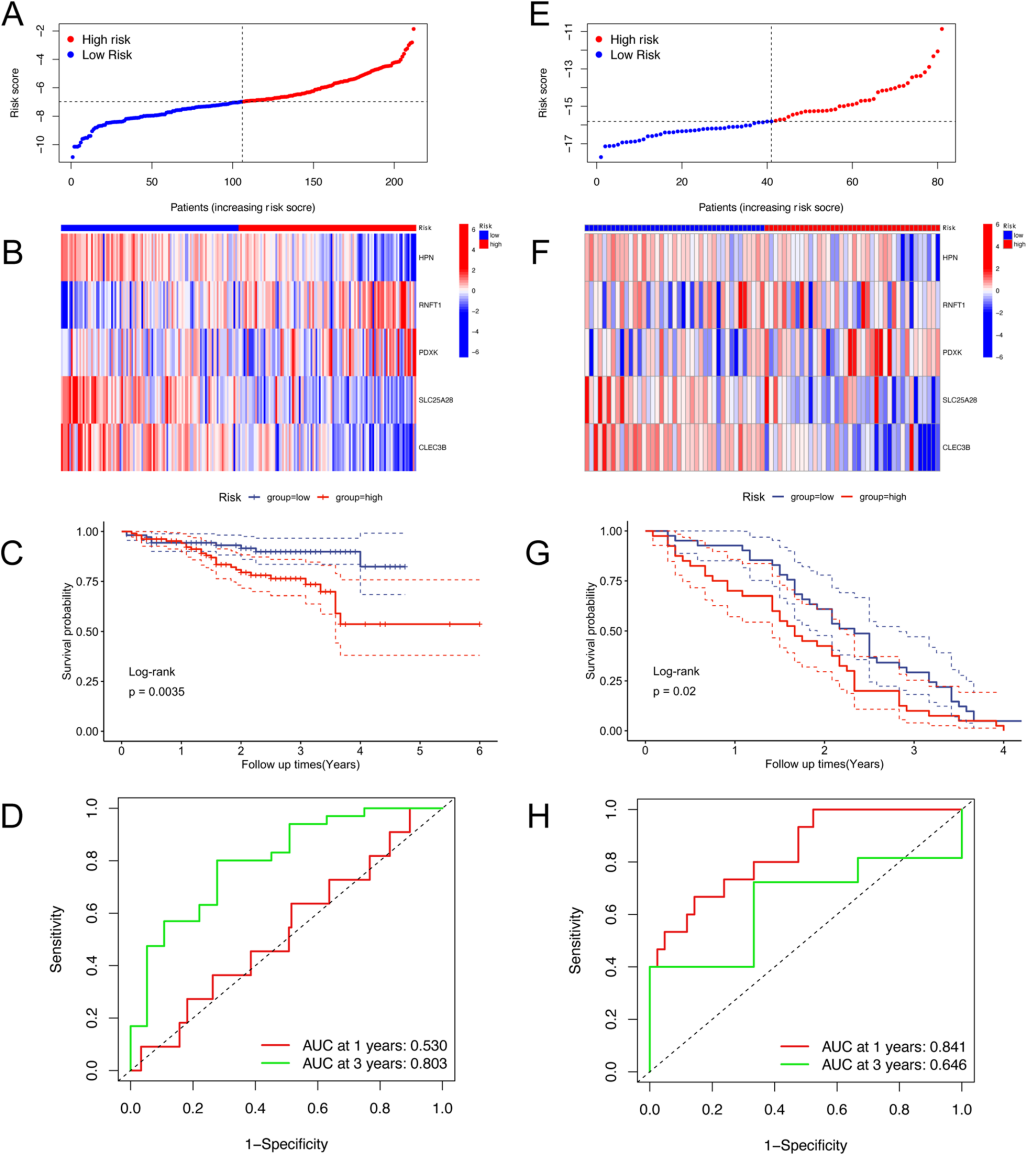
Verification of the risk model in external cohorts. **A**–**C**, **E**–**G** The predictive value of the cuproptosis-related genes signature was validated using the external cohorts of ICGC (**A**–**C**), and GSE54236 (**E**–**G**). **D**, **H** The survival ROC curves were used to assess the predictive efficacy of a 1- and 3-year overall survival in ICGC (**D**), GSE54236 (**H**). AUC: area under the curve

To further validate the predictive value of this 5 gene signature, the cohorts of ICGC and GSE54236 were extracted. Risk score of patients in these external validation cohort were calculated. Using the median cut-off value of the risk score, patients were stratified into high and low risk group. The external cohort results of survival analysis confirmed the robustness and reliability of the predictive values of this signature. That is, in the ICGC (Fig. 4A–C), and GSE54236 (Fig. 4E–G) cohorts, HCC patients in the high-risk group had a worse prognosis than those in the low-risk group. According to the ROC curve, the AUC of the cuproptosis-related genes signature for predicting 1- and 3-years overall survival was 0.530 and 0.803 in ICGC (Fig. 4D), respectively, and 0.841 and 0.646 in GSE54236, respectively (Fig. 4H).

### The construction and validation of the nomogram

**Fig. 5:**
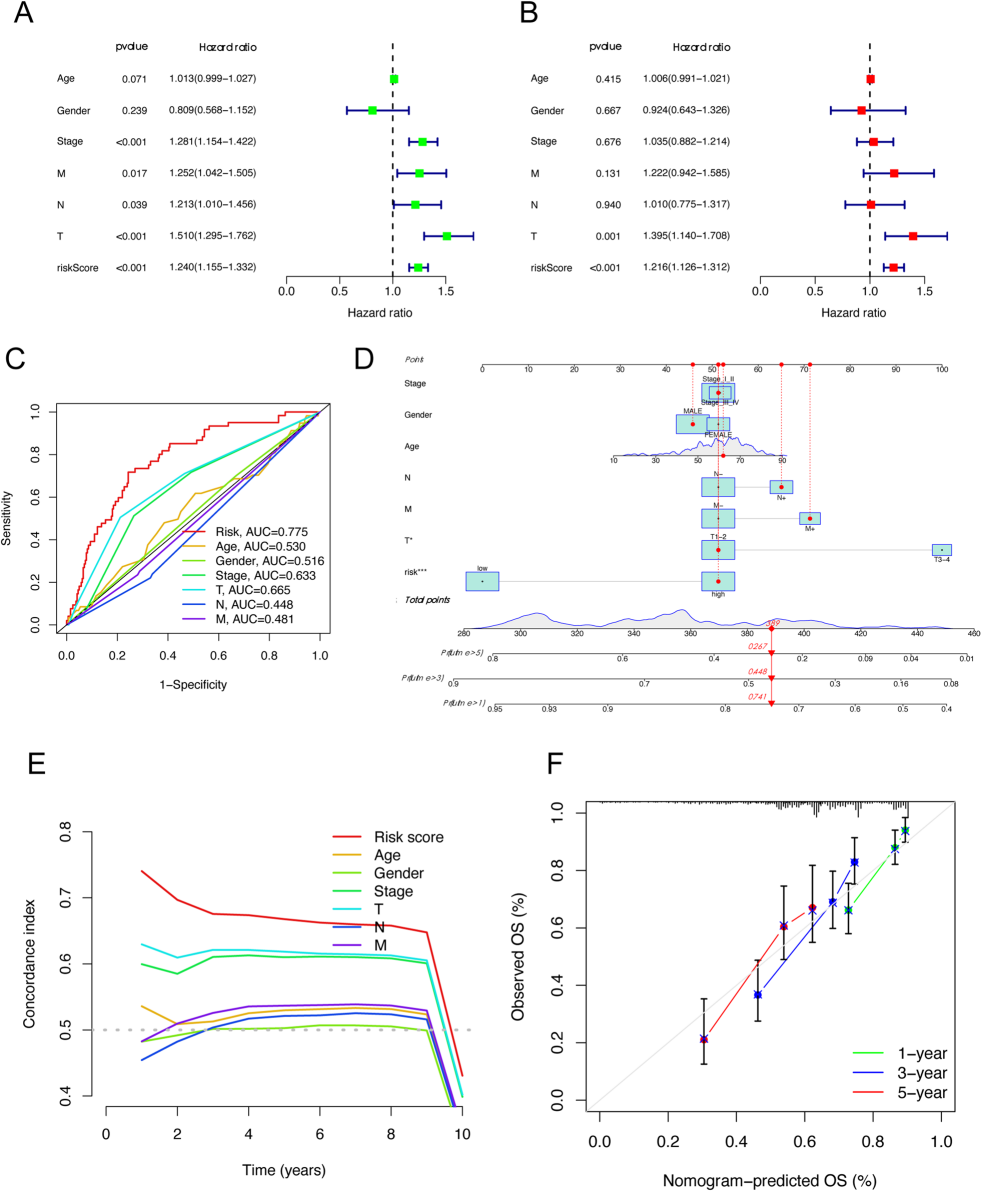
Construction and validation of Nomogram for predicting survival probability of HCC. **A, B** The univariate and multivariate cox regression analyses for identification of prognostic risk factors among the risk score and clinicopathological items (age, gender, T stage, N stage, M stage, and NCCN stage). **C** The AUC of survival ROC curves was utilized to assess the prognostic prediction efficacy of the established signature and clinicopathology items. **D** A nomogram for predicting the overall survival of HCC. **E** A concordance index (C-index) was generated to assess the identification and forecasting capabilities of the nomogram. **F** The calibration chart was used to assess the consistency between the predicted OS and the observed OS

Further, the univariate cox regression analysis was applied to see if the risk score and clinicopathological items (age, gender, T stage, N stage, M stage, and NCCN stage) were prognostic predictors. The results showed that the risk score (HR = 1.240, 95% CI 1.155–1.332), T stage (HR = 1.510, 95% CI 1.295–1.762), and N stage (HR = 1.213, 95% CI 1.010–1.456), M stage (HR = 1.252, 95% CI 1.042–1.505), and NCCN stage (HR = 1.281, 95% CI 1.154–1.422), were substantially linked with the OS of HCC patients (Fig. 5A). Furthermore, the multivariate cox regression analyses revealed that the risk score (HR = 1.216, 95% CI 1.126–1.312) and T stage (HR = 1.395, 95% CI 1.140–1.708) were the independent risk factor for OS (Fig. 5B). Meanwhile, the survival ROC analysis showed that the AUC was 0.775, 0.665, and 0.633 for risk score of the signature, T stage and the NCCN stage, respectively (Fig. 5C).

Additionally, a nomogram was constructed using the risk score, age, gender, T stage, N stage, M stage, and NCCN stage to precisely predict a 1-, 3-, and 5-year survival in HCC patients, where a higher total score indicated worse survival (Fig. 5D). The five cuproptosis-related genes-based signatures contributed the most to OS in HCC, according to the nomogram. Furthermore, the NCCN stage and the cuproptosis-related genes signature model had a high differentiating capacity, according to the concordance index (C-index) (Fig. 5E). The calibration curve revealed that there was a high consistence between the predicted OS and the observed OS of a 1-, 3-, and 5-year, which indicated high predictive accuracy of the cuproptosis-related gene signature (Fig. 5F).

### Association of cuproptosis-related genes signature with tumor immune microenvironment

**Fig. 6:**
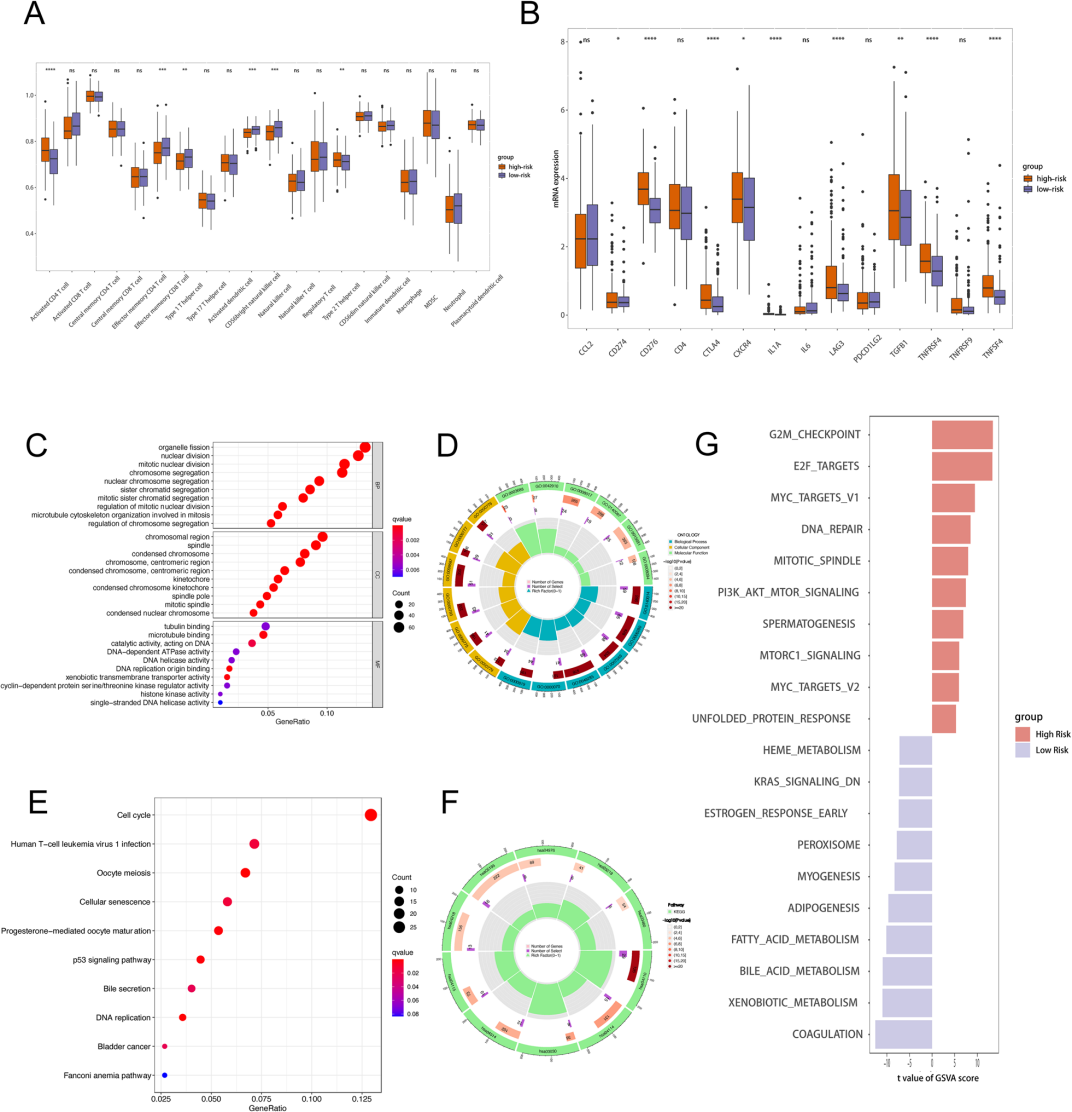
The tumor immune infiltrations and the annotation of differentially expressed genes in different risk groups based on the risk model. **A** The boxplot demonstrated the results of ssGSEA between low-risk and high-risk groups. **B** The boxplot showed the expression of immune checkpoint between low-risk and high-risk groups. **C**, **D** The GO annotation results of differentially expressed genes between low-risk and high-risk groups, including the results of biological processes (BP), molecular functions (MF), and cellular components (CC). **E**–**G** The KEGG and GSVA enrichment results of differentially expressed genes between different risk groups

The “ssGSEA” method was used to estimate immune cell infiltration. We discovered in the high-risk group, a greater number of active CD4 + T cells and Type2 T helper cell were enriched. In contrast, the effector memory CD8 T cell, Type1 T helper cell, CD56bright natural killer cell and Natural killer cell were shown to be considerably enriched in patients with a low risk (Fig. 6A). In addition, immune checkpoint inhibitor expression was significantly higher in HCC with high risk, particularly CD274, CD276, CD4, CTLA4, CXCR4, IL1A, LAG3, TGFB1, TNFRSF4, TNFRSF9 and TNFSF4 (Fig. 6B).

### Functional analysis for cuproptosis-related prognostic signature

The differentially expressed genes between high-risk and low-risk patients were mostly enriched in nuclear division, mitotic nuclear division, chromosome segregation, etc. in BP, chromosomal region, spindle, condensed chromosome, etc. in CC, and tubulin binding, microtubule binding and catalytic activity acting on DNA, etc. in MF, according to GO annotation (Fig. 6C, D). These differentially expressed genes were shown to be involved in cell cycle, cellular senescence, and p53 signaling pathway, etc. according to the KEGG data (Fig. 6E, F). In the high-risk group, the cuproptosis-related genes signature activated G2M checkpoint, E2F target, and MYC target and DNA repair signaling, according to the GSVA analysis of cancer hallmarks. The cuproptosis-related genes signature, on the other hand, triggered Coagulation, Xenobiotic metabolism, Bile acid metabolism, and Fatty acid metabolism in the low-risk group (Fig. 6G).

### PDXK was highly expressed in HCC specimens

**Fig. 7:**
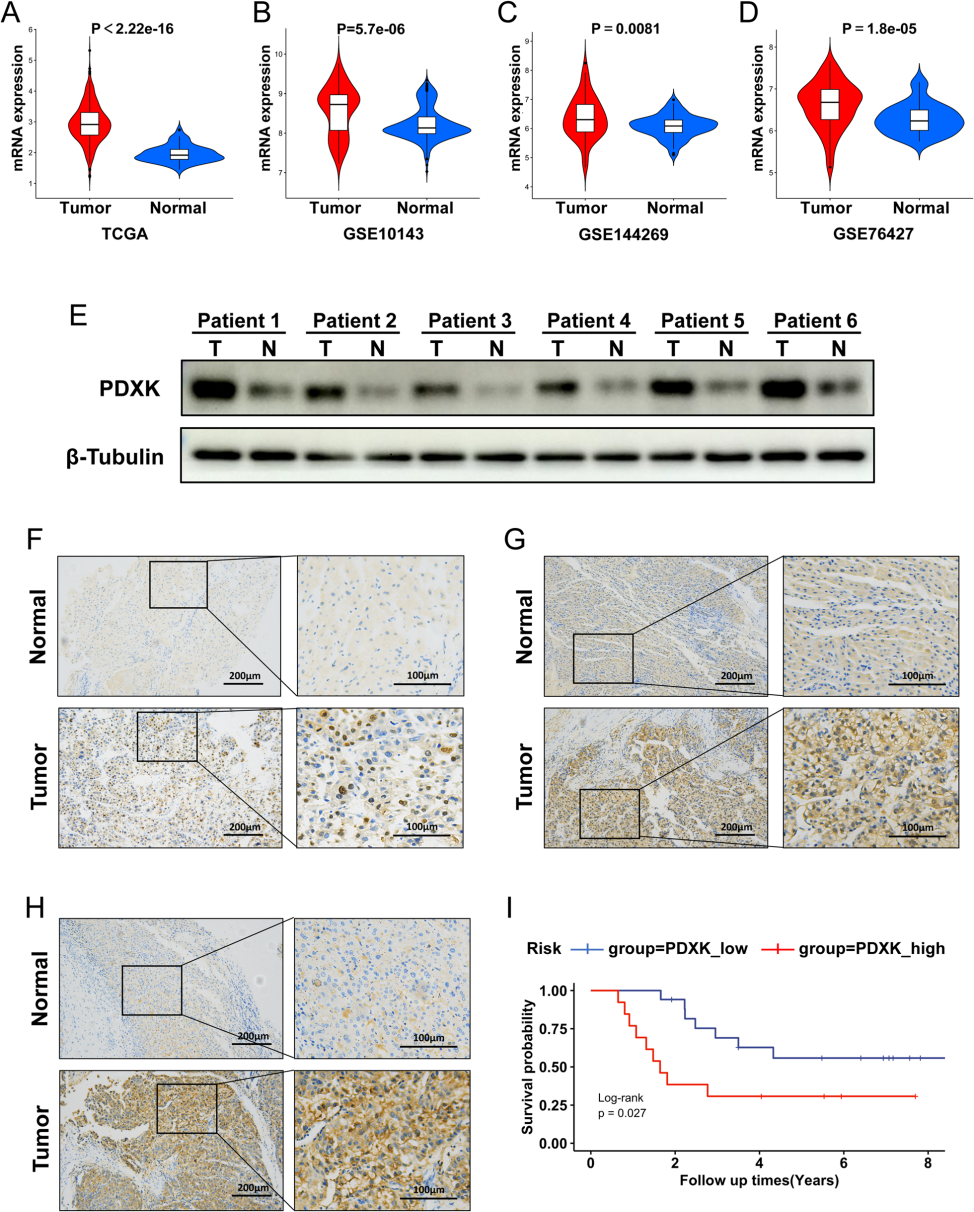
The relative expression of PDXK in tumor and normal samples. **A**–**D** the violin plot depicts the upregulation of PDXK in HCC tumor samples in TCGA, GSE10143, GSE144269, and GSE76427. **E** The protein level of PDXK were tested in six tumor samples with their paired normal tissue. **F**–**H** Reprehensive image of the expression of PDXK in tumor and paired normal samples revealed by IHC. **I** The survival analysis showed the high PDXK expression indicated a worse survival in our cohorts

After literature review, we found that among five cuproptosis-related gene, the role of PDXK in HCC remained unclear. Therefore, we further explore its expression pattern in HCC specimens. The expression of the PDXK was significantly higher in HCC tissues than in normal tissues in both the TCGA, GSE10143, GSE144269, and GSE76427 (Fig. 7A–D). PDXK was also found to be upregulated in HCC tumor samples compared to the paired normal tissue by Western Blot (Fig. 7E). Furthermore, IHC was used to investigate the protein level of PDXK in 30 paired HCC tumor and normal specimens. Results suggested that the expression of PDXK was upregulated in the HCC cancer samples compared with para-tumor normal samples (Fig. 7F–H). Totally, 30 HCC specimens were classified as high- and low-PDXK expression. We analyzed the relationship between PDXK expression and clinicopathological features of HCC patients. No significant correlation was found between PDXK expression and clinicopathological features such as tumor size, tumor grade, BCLC staging, TNM staging and etc. (Table 1). Besides, the univariate Cox regression analysis identified five features (BCLC stage, portal vein tumor thrombus, TNM stage, tumor count, and PDXK expression) that were significantly associated with survival in clinical cohorts (Table 2). As a result of the multivariate analysis, higher TNM stage and higher PDXK expression were considered to be independent risk factors for OS of HCC patents (Table 2). Kaplan–Meier analysis revealed that high expression of PDXK indicated a worse survival in our clinical cohort (Fig. 7I).

**Table 1:** Correlation of PDXK expression with different clinicopathological items of 30 patients with HCC in our clinical cohort

	PDXK Low (N = 17)	PDXK High (N = 13)	*P*-value
*Gender*			
Female	2 (11.8%)	1 (7.7%)	1
Male	15 (88.2%)	12 (92.3%)	
*Age (years)*			
Mean (SD)	47.9 (12.5)	51.6 (7.78)	0.333
Median [Min, Max]	47.0 [30.0, 74.0]	50.0 [40.0, 65.0]	
*AFP*			
< 20	8 (47.1%)	7 (53.8%)	1
> 20	9 (52.9%)	6 (46.2%)	
*HBsAg*			
Mean (SD)	0.765 (0.437)	0.846 (0.376)	0.588
Median [Min, Max]	1.00 [0, 1.00]	1.00 [0, 1.00]	
*Distant metastases*			
Mean (SD)	0.118 (0.332)	0.231 (0.439)	0.446
Median [Min, Max]	0 [0, 1.00]	0 [0, 1.00]	
*Tumor size (cm)*			
< 3	5 (29.4%)	2 (15.4%)	0.642
> 3	12 (70.6%)	11 (84.6%)	
*Tumor count*			
Mean (SD)	1.12 (0.485)	1.46 (0.967)	0.257
Median [Min, Max]	1.00 [1.00, 3.00]	1.00 [1.00, 4.00]	
*PVTT*			
Mean (SD)	0.118 (0.332)	0.308 (0.480)	0.236
Median [Min, Max]	0 [0, 1.00]	0 [0, 1.00]	
*Cirrhosis*			
Mean (SD)	0.529 (0.514)	0.385 (0.506)	0.448
Median [Min, Max]	1.00 [0, 1.00]	0 [0, 1.00]	
*BCLC stage*			
A	13 (76.5%)	8 (61.5%)	0.665
B	1 (5.9%)	1 (7.7%)	
C	3 (17.6%)	4 (30.8%)	
*TNM stage*			
I Stage	9 (52.9%)	5 (38.5%)	0.426
II Stage	4 (23.5%)	2 (15.4%)	
III Stage	4 (23.5%)	6 (46.2%)	
*Tumor grade*			
2	9 (52.9%)	2 (15.4%)	0.104
3	7 (41.2%)	10 (76.9%)	
4	1 (5.9%)	1 (7.7%)	

*PVTT* Portal vein tumor thrombus

**Table 2:** Cox regression analysis of OS in 30 patients with HCC in our clinical cohort

	Univariate analysis			Multivariate analysis		
	HR	95% CI	*P*-value	HR	95% CI	*P*-value
Age	1.01	0.97–1.06	0.598	–	–	–
Gender	1.21	0.16–9.21	0.852	–	–	–
HBsAg	0.75	0.24–2.32	0.614	–	–	–
Cirrhosis	0.47	0.16–1.34	0.157	–	–	–
AFP	1.59	0.59–4.26	0.361	–	–	–
Tumor Size	2.49	0.56–11	0.228	–	–	–
Tumor Count	2.21	1.14–4.28	0.019	1.25	0.61–2.55	0.5415
PVTT	2.97	1.01–8.79	0.049	2.59	0.29–23.43	0.3965
Distant Metastases	0.78	0.18–3.45	0.747	–	–	–
TNM Stage	3.96	1.98–7.91	0	5.57	2.32–13.36	1.00E–04
BCLC Stage	1.74	1.04–2.93	0.035	0.59	0.2–1.72	0.3314
Tumor Grade	2.04	0.93–4.47	0.075	–	–	–
PDXK	2.92	1.08–7.92	0.035	3.98	1.18–13.41	0.026

*PVTT* Portal vein tumor thrombus

Correlation analysis of TCGA-LIHC dataset showed that the expression of PDXK was positively correlated with CD3, CD4, CD8, CD20 and CD68 (Additional file 4: Fig. S1A–E). Similarly, the results from IHC demonstrated that the protein levels of CD3, CD4, CD8, CD20 and CD68 were higher in patients with a high protein level of PDXK, which indicated a higher infiltration of inflammatory cells in these patients including CD4 + lymphocyte, CD8 + lymphocyte, B lymphocyte, and macrophages (Additional file 4: Fig. S1F).

### Knockdown of PDXK inhibited cell proliferation and metastasis of HCC

**Fig. 8:**
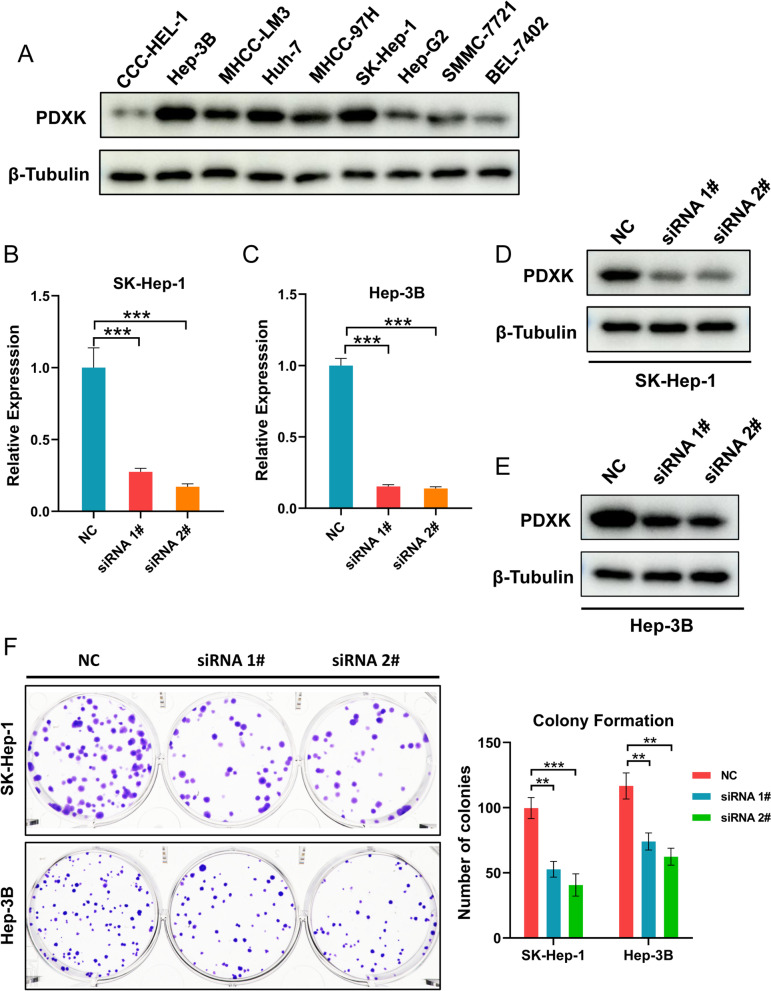
PDXK promote cell proliferation in HCC. **A** The expression of PDXK in multiple LICH cancer cell lines were performed. The Hep-3B and SK-Hep-1, which showed the highest expression of PDXK, were chose for further in vitro experiments. **B**–**E** The efficiency of silencing PDXK was indicated by Western blot and RT-qPCR in SK-Hep-1and Hep3B cell lines. **F** Knockdown of PDXK inhibited the proliferation ability of SK-Hep-1 and Hep-3B cell were evaluated by a Colony assay

**Fig. 9:**
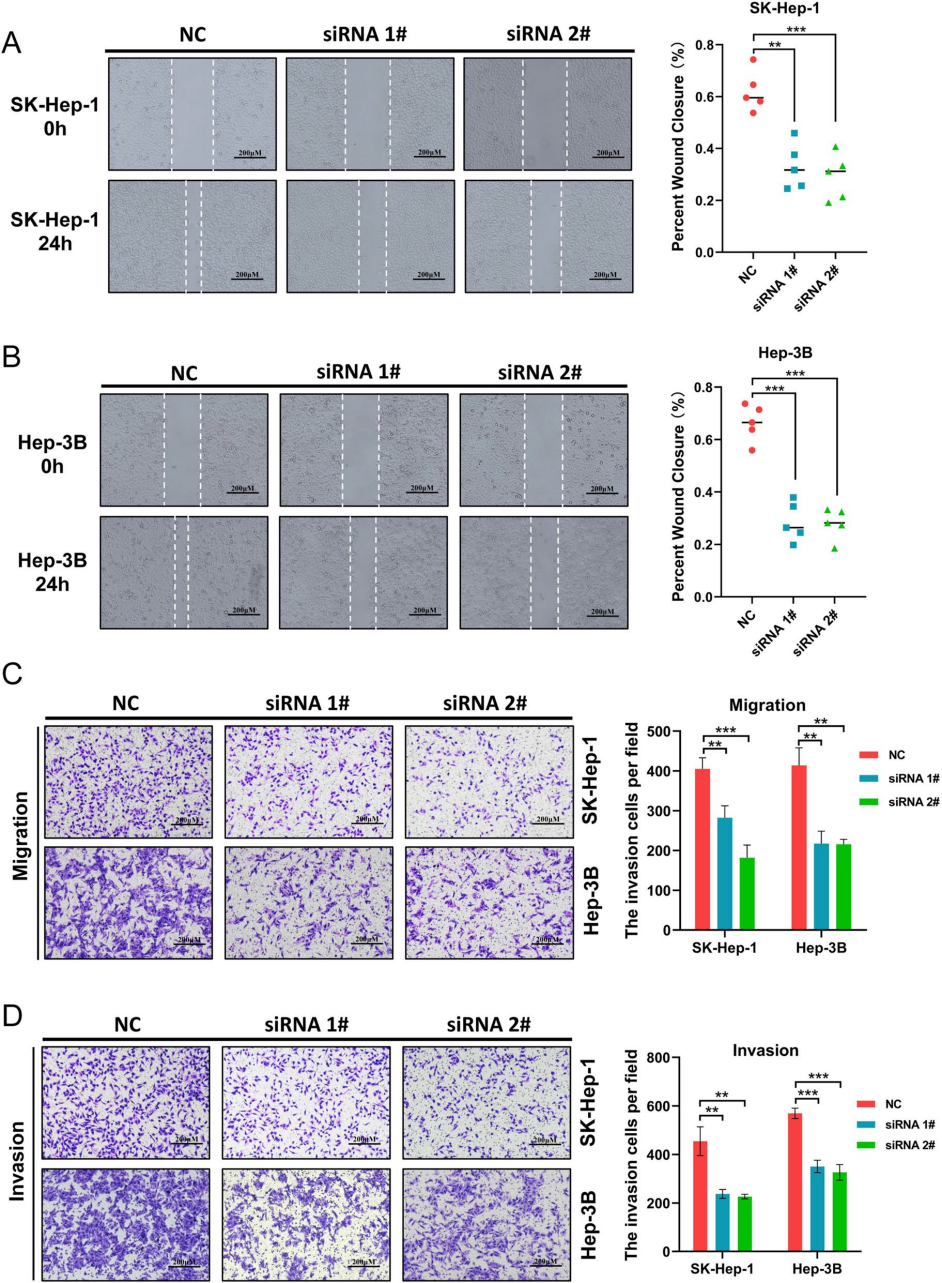
PDXK promote cell migration and invasion in HCC. **A**, **B** The wound healing assay showed that PDXK knockdown inhibited the migration of HCC tumor cell lines of SK-Hep-1 and Hep-3B. **C** The transwell migration assay showed that PDXK knockdown compromised the migration ability of SK-Hep-1 and Hep-3B. **D** The transwell invasion assay showed that PDXK knockdown attenuated the invasion ability of SK-Hep-1 and Hep-3B. Data are presented as mean ± standard error of at least three independent experiments

After we validated the expression pattern of PDXK in HCC specimens, we also explored its function in HCC cells. The expression of PDXK was detected in multiple LICH cancer cell lines, including CCC-HEL-1, Hep-3B, MHCC-LM3, Huh-7, MHCC-97H, SK-Hep-1, Hep-G2, SMMC-7721, and BEL-7402. Hep-3B and SK-Hep-1 cells had relatively high expression of PDXK than other cells (Fig. 8A). Thus, Hep-3B and SK-Hep-1 were used for further functional experiments to explore the role of PDXK in HCC. The efficiency of silencing PDXK in Hep-3B and SK-Hep-1 was validated using RT-qPCR (Fig. 8B, C) and Western blot (Fig. 8D, E). Colony formation assays revealed that the knockdown of PDXK suppressed cell proliferation (Fig. 8F). The wound healing assay then revealed that the inhibition of PDXK significantly suppressed cell migration of SK-Hep-1 (Fig. 9A) and Hep-3B (Fig. 9B). The transwell migration assay demonstrated that PDXK knockdown significantly attenuated the migration ability of Hep-3B and SK-Hep-1(Fig. 9C), and the invasion ability of Hep-3B and SK-Hep-1 were also compromised after knockdown of PDXK (Fig. 9D). Therefore, the knockdown of PDXK could significantly suppress the proliferation and metastasis of HCC cells.

### PDXK deficiency enhances the sensitivity of HCC cells to cuproptosis agonists

**Fig. 10:**
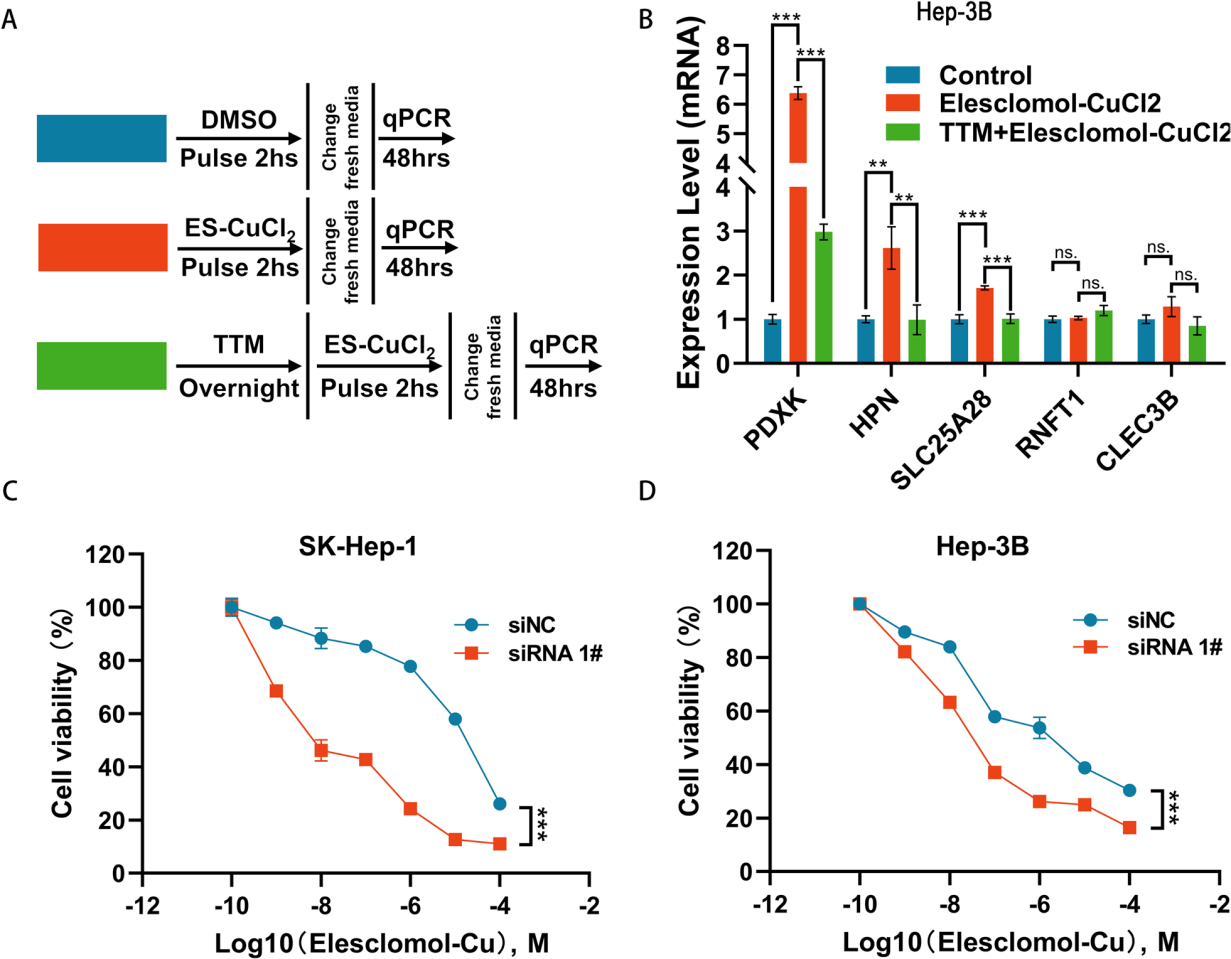
PDXK is related to copper ionophore-induced cell death. **A** The illustration of cuproptosis induction model. **B** The expression level change of cuproptosis-related genes (PDXK, HPN, SLC25A28, RNFT1 and CLEC3B) in indicated groups were presented by qPCR. **C**, **D** HCC cells transfected with PDXK specific siRNA or scramble control were treated with the indicated concentrations of elesclomol-CuCl_2_ and cell viability was evaluated by MTT assay after 72 h. ns, not significant, *p* > 0.05; *p < 0.05; ***p < 0.001. TTM, tetrathiomolybdate

By constructing a cuproptosis induction model, we further explored whether the expression of promising related genes are affected by cuproptosis (Fig. 10A). This model includes three experimental groups: negative control, Elesclomol-CuCl_2_, and TTM-Elesclomol-CuCl_2_. In the Elesclomol-CuCl_2_ group, Hep-3B cells were treated with 30 nM Elesclcomol-CuCl_2_ (1:1, Elesclomol is a potent copper ionophore which induce cuproptosis) for 2 h; In the TTM-Elesclomol-CuCl_2_ group, Hep-3B cells were pretreated with 20 μM tetrathiomolybdate (TTM, a copper ion chelator that inhibits cuproptosis) overnight and then treated with 30 nM Elesclomol-CuCl_2_ for 2 h. The treated cells were cultured in fresh medium for 48 h and then RNA was extracted and gene expression of cuproptosis-related genes (PDXK, HPN, SLC25A28, RNFT1 and CLEC3B) was detected by qPCR. The results showed that the expression of PDXK, HNP and SLC25A28 were upregulated in Hep-3B cells after Elesclomol-CuCl_2_-induced cuproptosis; however, the upregulation was inhibited after pretreatment with TTM (Fig. 10B).

To further clarify the role of PDXK in cuproptosis, the cell viability was examined after the treatment of Elesclomol-CuCl_2_ in PDXK deficient Hep-3B and SK-Hep-1 cells using MTT assay. The results revealed that the sensitivity of HCC cells to cuproptosis agonist was increased after knockdown of PDXK (Fig. 10C, D). These results thus demonstrated that the deficiency of PDXK facilities cuproptosis in HCC cells.

## Discussion

Recently, Tsvetkov et al*.* demonstrated a new kind of cell death, named cuproptosis, which is initiated by high Cu-induced mitochondrial protein lipoylation including DLST, DLAT, DBT, and GCSH. Toxic lipoylated protein buildup caused the cellular loss of Fe-S cluster proteins, promoted proteotoxic stress, and ultimately resulted in cell death [4]. BSO, a potent inhibitor of γ-glutamylcysteine synthetase, significantly increased cuproptosis susceptibility in cancer cells [4]. It indicated that cuproptosis translational medicine appears to be a promising candidate for clinical applications in a variety of human cancers.

In this research, five cuproptosis-related genes (HPN, RNFT1, PDXK, SLC25A28, and CLEC3B) were chosen to construct a prognostic signature based on their performance in the LASSO cox regression analysis. The signature’s prognostic values were validated using two independent datasets (ICGC and GSE54236). The findings suggested that this signature could be a more accurate predictor of patient prognosis. Among the five signature genes, the high expression of PDXK in HCC tumor samples were confirmed by the Western Blot and IHC, and the high expression of PDXK indicated a worse survival in our clinical cohorts. PDXK is involved in the metabolism of vitamin B6 from the non-phosphorylated form pyridoxal (PL), pyridoxine (PN), and pyridoxamine (PM) to the phosphorylated form pyridoxal phosphate (PLP), pyridoxine phosphate (PNP), and pyridoxamine phosphate (PMP) [19]. It was reported that acute myeloid leukemia (AML) relies on PDXK kinase activity and PDXK inhibition suppresses the proliferation of AML cells [20]. PDXK was highly expressed in ovarian cancer tissues and cells, and high PDXK expression was positively correlated with poor differentiation, advanced FIGO stage, and worse prognosis. Mechanistically, PDXK knockdown significantly inhibited the tumor proliferation by retaining the cells in the G0/G1 phase [21]. In our research, we found that the metastasis and proliferation of HCC cancer cells were compromised by the knockdown of PDXK. Furthermore, the expression of PDXK was upregulated after the induction of cuproptosis, which could be suppressed when pretreated with a copper ion chelator. And silencing of PDXK increased the sensitivity of HCC cells to cuproptosis. These results indicated that PDXK is evolved in cuproptosis, making PDXK a novel target that sensitizes tumor cells to cuproptosis inducers. Nonetheless, additional research is needed to determine how PDXK modulates cuproptosis.

Regarding the other genes among the signature genes, HPN encodes a type II transmembrane serine protease, which may cleaves extracellular substrates and helps with the proteolytic processing of growth factors like HGF and MST1/HGFL and may be associated with the proliferation and progression of cancer [22]. It is predicted that SLC25A28 will facilitate the activity of the ferrous iron transmembrane transporter and play a role in iron import into the mitochondrion. Zhang et al*.* [23] reported that ferroptosis events mediated by BRD7 or p53 were compromised by SLC25A28 knockdown in hepatic stellate cells. The protein encoded by RNFT1 is E3 ubiquitin-protein ligase that acts in the endoplasmic reticulum (ER)-associated degradation (ERAD) pathway, which ubiquitinates misfolded proteins that accumulate in the ER for subsequent proteasome-mediated degradation. The previous research indicated that RNFT1 may participate in the migration of breast cancer [24, 25]. The protein encoded by CLEC3B may be related to calcium ion binding and heparin binding activity. CLEC3B is downregulated in clear cell renal cell carcinoma (ccRCC) [26], lung adenocarcinoma (LUAD) [27] and HCC [28]. And the down-regulation of CLEC3B facilitates the proliferation of ccRCC cell lines through mitogen‑activated protein kinase pathway [26] and increases the epithelial-mesenchymal transition, migration, and invasion of LUAD and HCC cells [27, 28].

The relationship between cuproptosis and immune cell infiltration in HCC is also unknown. We revealed that higher infiltrations of immune cells including effector memory CD8 T cell, Type1 T helper cell, CD56bright natural killer cell, and Natural killer cell were observed in the low-risk group. In contrast, higher infiltrations of activated CD4 + T cells and Type2 T helper cells were observed in the high-risk group. Furthermore, HCC in the high-risk group based on the cuproptosis-related signature tended to have lower levels of immune checkpoint molecule expression including CD274, CD276, CD4, CTLA4, CXCR4, IL1A, LAG3, TGFB1, TNFRSF4, and TNFSF4, indicating a reduced likelihood of benefiting from immune checkpoint inhibitors.

There are few flaws in our research. Firstly, the prognostic model was obtained based on the analysis of public datasets, which still needs to be validated with prospective, multicenter cohorts. Secondly, only one of the risk signature genes, PDXK, was explored by the in vitro experiments. The function of HPN, SLC25A28, RNFT1, and CLEC3B in the carcinogenesis and progression of LICH need to be further explored. Lastly, experimentation on the basic mechanisms of how cuproptosis-related genes regulate cuproptosis is required.

## Supplementary Information

**Additional file 1: Table S2.** The list of antibody and dilution ratio.

**Additional file 2: Table S3.** The list of primer sequences involved in qPCR.

**Additional file 3: Table S1.** The expression correlation of the cuproptosis-related genes and the cuproptosis genes.

**Additional file 4: Fig. S1.** The correlation of PDXK expression and markers of inflammatory cells. **A**–**E** Correlation analysis of TCGA-LIHC dataset showed that the expression of PDXK was positively correlated with CD3, CD4, CD8, CD20 and CD68. **F** Reprehensive images of IHC showed the protein level of CD3, CD4, CD8, CD20 and CD68 in patients with different protein level of PDXK.

## Data Availability

The study’s original contributions are included in the article/Additional files 1, 2, 3, and 4; further inquiries should be directed to the corresponding author.
