# Supplementary material for: Identification of a prognostic cuproptosis-related signature in hepatocellular carcinoma
Source: Biol Direct. 2023 Feb 7;18:4. doi: 10.1186/s13062-023-00358-w (PMC9903524; doi:10.1186/s13062-023-00358-w)
Supplement: Supplementary file 1 — Additional file 1: Table S2. The list of antibody and dilution ratio. [file 13062_2023_358_MOESM1_ESM.pdf]

**Supplementary Table 2.** The list of antibody and dilution ratio

| Antibody | Manufacturer | Cat. NO | Dilution |
|---|---|---|---|
| PDXK | Proteintech | 15309-1-AP | WB: 1:1000<br>IHC: 1:200 |
| β-Tubulin | Servicebio | GB11017 | WB: 1:2000 |
| CD3 | Abcam | ab16669 | IHC: 1:200 |
| CD4 | Abcam | ab183685 | IHC: 1:500 |
| CD8 | Abcam | ab217344 | IHC: 1:2000 |
| CD20 | Proteintech | 17329-1-AP | IHC: 1:2000 |
| CD68 | boster | BA3638 | IHC: 1:400 |
| Goat anti-Rabbit HRP | Proteintech | SA00001-2 | WB: 1:10000 |
