# Supplementary material for: Identification of a prognostic cuproptosis-related signature in hepatocellular carcinoma
Source: Biol Direct. 2023 Feb 7;18:4. doi: 10.1186/s13062-023-00358-w (PMC9903524; doi:10.1186/s13062-023-00358-w)
Supplement: Supplementary file 2 — Additional file 2: Table S3. The list of primer sequences involved in qPCR. [file 13062_2023_358_MOESM2_ESM.pdf]

Supplementary Table 3. The list of primer sequences involved in qPCR

Gene	Primer sequences
PDXK-Forward	GATTTGAGATTGACGCGGTGA
PDXK-Reverse	CCCTCGTATAACCTGTGAGCAC
SLC25A28-Forward	GTACCCCATCGACTGCGTC
SLC25A28-Reverse	CTCCAACACATTGCGATAGCG
HPN-Forward	GGGCCATTGTGGCTGTTCT
HPN-Reverse	CGTCCCTTCCGTCTTGTCAAA
RNFT1-Forward	CCTGAAGCAAAGACATCTGGG
RNFT1-Reverse	ACTGTGCAGTTGGCTACGATT
CLEC3B-Forward	CCCAGACGAAGACCTTCCAC
CLEC3B-Reverse	CGCAGGTACTCATACAGGGC
