# Supplementary figures and images for: Identification of a prognostic cuproptosis-related signature in hepatocellular carcinoma
Source: Biol Direct. 2023 Feb 7;18:4. doi: 10.1186/s13062-023-00358-w (PMC9903524; doi:10.1186/s13062-023-00358-w)

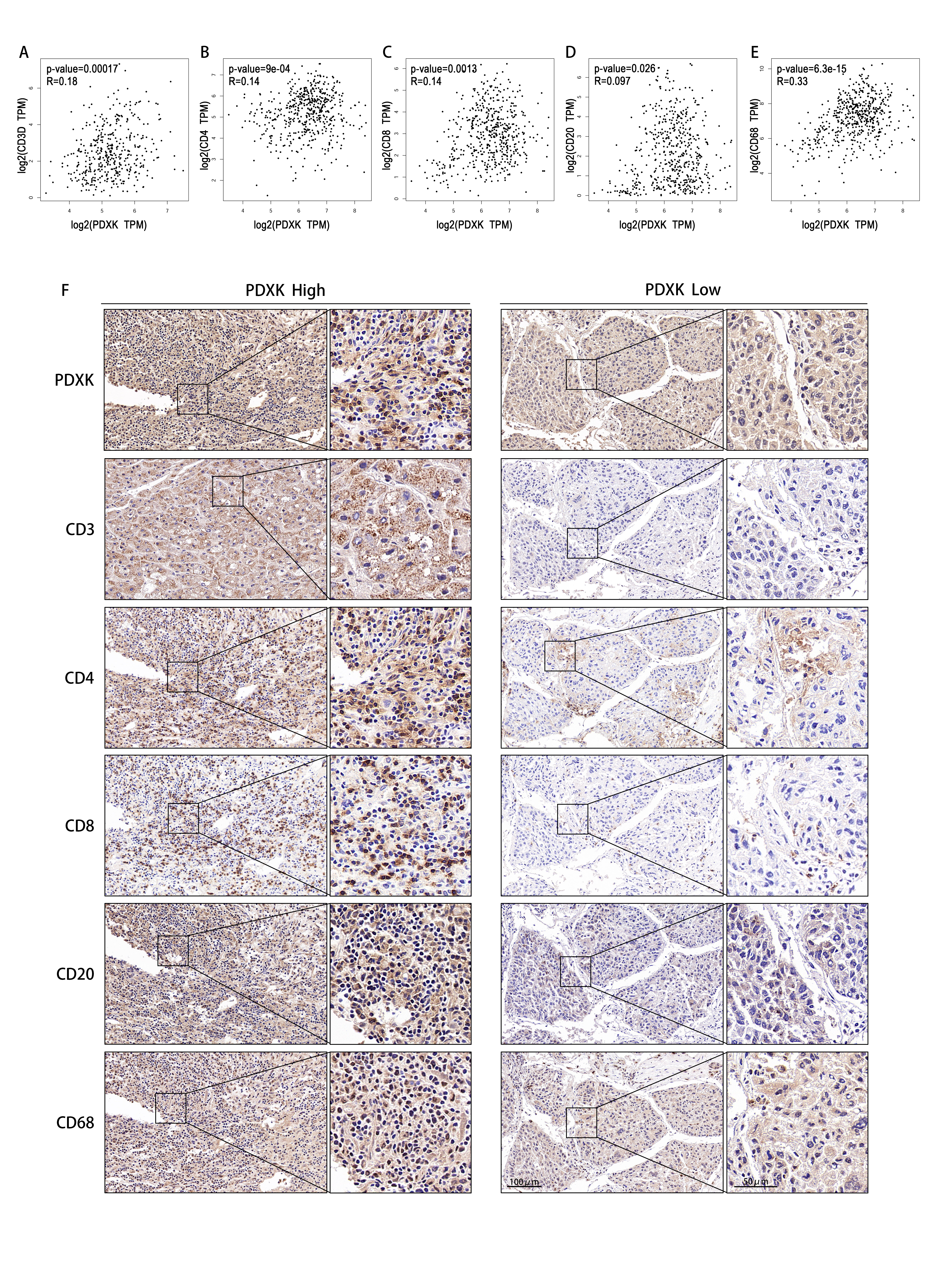

Supplement: Supplementary file 4 — Additional file 4: Fig. S1. The correlation of PDXK expression and markers of inflammatory cells. A–E Correlation analysis of TCGA-LIHC dataset showed that the expression of PDXK was positively correlated with CD3, CD4, CD8, CD20 and CD68. F Reprehensive images of IHC showed the protein level of CD3, CD4, CD8, CD20 and CD68 in patients with different protein level of PDXK. [file 13062_2023_358_MOESM4_ESM.tif]
